# Remarks, Gleanings, and Extracts

**Published:** 1874-05

**Authors:** S. M. Burnett

**Affiliations:** Tennessee


### REMARKS, GLEANINGS AND EXTRACTS

BY S. M. BURNETT, OF TENNESSEE.

*Test for Blood.*—Dr. Bertholet gives, in the *Amer. Jour. of Med. Sciences,* a very interesting article on the Guaiacum process for the detection of blood as a valuable aid in distinguishing nucleated from non-nucleated blood disks.

The blood of mammals differs from that of other classes of animals in being non-nucleated and in being circular. The disks of the camel and archenia are elliptical but they have no nucleus. It is often of great importance in medico legal cases to determine from what animal blood is derived, and this can only be done by an examination of the disks. The blood in these cases is nearly always dried, and to restore the shrunken cerpucles to their original form is impossible—hence the presence of a nucleus is the only point left on which a positive assertion can be founded. The Guaiacum process is the most reliable for demonstrating the presence of the nucleus that we have. The following method of preceedure is that recommended by Dr. Bertolet: "If a microscopic preparation, mounted in feebly acidulated glycerine or other suitable fluid, is carefully irrigated with a properly prepared alcoholic solution of Guaiacum resin; then when a very small quantity of etherial solution of peroxide of hydrogen (ozonic ether) is introduced beneath the glass cover, a bluing varying irom a light sapphire to a deep indigo shade, will be observed in the several corpuscles of mammalian blood. Though the degree of coloration of the different corpuscles presents considerable variety, yet all parts of the individual corpuscle itself are colored uniformly. If the blood has been derived from an animal having *nucleated* corpuscles, an *entirely different* picture is presented; when treated by the same reagents the *nucleus is seen as a sharply defined, dark blue body, while the protoplasm surrounding it assumes a mere delicate violet hue.* Some times the other parts of the blood globulous are not the least colored; in all cases however, the nucleus is deeply stained and easily recognizable when present even by the naked eye."

*According* to the latest researches the poisonous action of theine and caffiene is very largely expended upon the circulatory system, first stimulating and afterwards paralyzing the nerves of the vasomotor system. This is soon reflected to other portions of the system and we have palpitation, irregular cardiac active, and various nervous symptoms. All this, to say nothing of the effects of the tannin and hot water on the stomach, lurks in the cup that proverbially "cheers but don't inebriate."

*Action of Alcohol as a Therapeutic Agent.*—In a classical article on "tolerance of disease" in the January number of the *American Practitioner*. Prof. Austin Flint, answers in a nutshell the question. What is the action of alcohol as a therapeutic agent? After giving as accurate a definition of what is meant by "tolerance of disease" as our present knowledge will allow and considering its influence upon the diagnosis and prognosis of disease, he treats of its application to the treatment of disease. In all those affections where the danger of death is from asthenia, it is the duty of the practitioner to husband this tolerance and to avoid all those measures calculated to impair it. "Tonic remedies "he says," have a certain measure of importance as conducive to tolerance. Alcohol has its place of usefulness here. Without discussing the question whether alcohol be a food or a remedy, and divesting the topic of all moral considerations, that it is an important constituent of the supporting treatment must be admitted. Practically, its usefulness as such consists in this; it promotes for a time tolerance; its use is indicated where there is evidence of failure in tolerance; it is often wise to forestall, by its use, failure of tolerance; the urging with which it is indicated is proportionate to the degree of danger from failure of tolerance. The proof of its usefulness is the evidence of increased tolerance without any of the phenomina of alcoholism or excetation."

*Poisonous Analine Dyes.*—Mr. Wm. Ward, writes to the *Scientific American* that, after eating three or four inches of stick candy, of a red color, he was taken sick with burning pain in his stomach. He grew worse, and in three days was not able to walk about, suffering pain all the time. A physician treated it as a case of aniline poisoning, and under the treatment he rapidly recovered.

*Pea-Nut Oil.*—The oil obtained from the pea-nut or ground pea, of the Southern States, is said to be an excellent substitute for olive oil, especially for table use, as it keeps a long while without becoming rancid.

*Enemata of Bromid. Potass* have been recommended in vomiting of pregnancy, beginning with 92 grs. per day, and gradually increased till bromism is produced.

*A Good Wash for Chilblains* is sulphorous acid, 3 parts; glycerine, 1 part; water, 1 part.

*Another Remedy for Hooping-Cough.*—This time it is nitric acid, and its advocate is Dr. Berry, in the *London Times and Gazette.* He gives it in doses of from five to ten minims of the dilute acid every three or four hours.

